# Genomic surveillance detects *Salmonella enterica* serovar Paratyphi A harbouring *bla*_CTX-M-15_ from a traveller returning from Bangladesh

**DOI:** 10.1371/journal.pone.0228250

**Published:** 2020-01-30

**Authors:** Satheesh Nair, Martin Day, Gauri Godbole, Tranprit Saluja, Gemma C. Langridge, Timothy J. Dallman, Marie Chattaway

**Affiliations:** 1 Gastrointestinal Bacteria Reference Unit, Public Health England, London, United Kingdom; 2 Department of Microbiology, City Hospital, Birmingham, United Kingdom; 3 Quadram Institute Bioscience, Norwich Research Park, Norwich, United Kingdom; 4 National Institute for Health Research Health Protection Research Unit, Gastrointestinal Infections, University of Liverpool, Liverpool, England; Tianjin University, CHINA

## Abstract

Whole genome sequencing (WGS) has been used routinely by Public Health England (PHE) for identification, surveillance and monitoring of resistance determinants in referred *Salmonella* isolates since 2015. We report the first identified case of extended-spectrum-β-lactamase (ESBL) *Salmonella enterica* serovar Paratyphi A (*S*. Paratyphi A) isolated from a traveller returning to England from Bangladesh in November 2017. The isolate (440915) was resistant to ciprofloxacin and harboured both the mobile element ISEcp9 –*bla*_*CTX-M-15*_-hp-tnpA and *bla*_TEM-191_, associated with ESBL production. Phenotypic resistance was subsequently confirmed by Antimicrobial Susceptibility Testing (AST). *S*. Paratyphi A 440915 harboured an IncI1 plasmid previously reported to encode ESBL elements in *Enterobacteriaceae* and recently described in a *S*. Typhi isolate from Bangladesh. Results from this study indicate the importance of monitoring imported drug resistance for typhoidal salmonellae as ceftriaxone is the first line antibiotic treatment for complicated enteric fever in England. We conclude that WGS provides a rapid, accurate method for surveillance of drug resistance genes in *Salmonella*, leading to the first reported case of ESBL producing *S*. Paratyphi A and continues to inform the national treatment guidelines for management of enteric fever.

## Introduction

Enteric fever is a systemic infection caused by the human adapted pathogens; *Salmonella enterica* serovar Typhi (*S*. Typhi) and *S*. *enterica* serovar Paratyphi (*S*. Paratyphi) A, B and C. Enteric fever continues to carry a burden of morbidity and mortality with an estimated ~28 million cases reported globally in 2000 [1]. *S*. Paratyphi A is the second most common cause of enteric fever after *S*. Typhi with approximately one *S*. Paratyphi A infection occurring for every four *S*. Typhi infections [1].

The greatest burden of illness is experienced by infants, children and adolescents in South-central and Southeast Asia [1]. As described for typhoid fever (caused by *S*. Typhi), paratyphoid fever is rare in industrialised countries although known to occur among travellers to endemic parts of the world [2][3]. In 2015, 18 EU/EEA countries reported a total of 845 confirmed cases, a notification rate of 0.23 cases per 100 000 population [4]. Between April 2015 and September 2018, Public Health England (PHE) reported an average of 115 cases in England and Wales of Paratyphoid A fever per year. The number of cases has been constant during this time period with a majority of cases acquired abroad; 54% were from travellers returning to the United Kingdom (UK) from the Indian subcontinent (https://www.gov.uk/government/publications/typhoid-and-paratyphoid-laboratory-confirmed-cases-in-england-wales-and-northern-ireland).

Antibiotic resistance in *S*. Paratyphi A is an emerging public health problem. Resistance to multiple first line antibiotics e.g. ampicillin, chloramphenicol and co-trimoxazole (multidrug resistance [MDR]) [5], nalidixic acid [6] and ciprofloxacin [7][8] has arisen in multiple countries since 2000. Alternative antimicrobial treatments, including third generation cephalosporins (ceftriaxone) or azithromycin, are increasingly used as first line therapies [9]. Recently, resistance to these newer drugs has been reported in enteric fever-causing salmonellae [10][11][12]. While these cases have largely been sporadic, a large-scale extensively drug resistant (XDR) *S*. Typhi outbreak began in Pakistan in 2016, harbouring resistance to third generation cephalosporins [13]. Resistance to ceftriaxone or other extended-spectrum ß-lactams is usually due to the production of extended-spectrum-ß-lactamases (ESBLs) of which *bla*_CTX-M_ type ESBLs are one of the determinants for cephalosporin resistance in *Salmonella* [14]. Many *bla*_CTX-M_ variants are described in the literature, with *bla*_CTX-M-9_, *bla*_CTX-M-14_ and *bla*_CTX-M-15_ being the most commonly reported [15][16][17]. *bla*_CTX-M_ type ESBLs are usually encoded by transmissible plasmids [18], hence routine surveillance of resistance determinants is essential to understand when and where populations may be affected.

WGS has been used at the PHE Gastrointestinal Bacterial Reference Unit (GBRU) since April 2015 for routine identification, surveillance, and detection of outbreak transmission events and Antimicrobial Resistance (AMR) determinants [3][19][20]. Through routine genomic surveillance in November 2017, we identified an ESBL-harbouring *S*. Paratyphi A strain isolated from a traveller returning to the UK from Bangladesh. Here we report the characterisation, location and composition of the region encoding ß-lactam resistance and suggest the possible transmission mechanism of this ESBL resistant *S*. Paratyphi A isolate imported into the UK.

## Materials and methods

### Case history

Enteric fever is a notifiable disease in the UK. Information was retrospectively collected from an enhanced surveillance questionnaire collected by the public health team from the case and hospital case notes.

### Bacterial isolate and phenotypic identification

A stool specimen submitted to the hospital in September 2017 was subjected to EntericBio, a rapid panbacterial PCR assay screening, and found to be *Salmonella* spp. PCR positive. The stool was cultured for *Salmonella* and the isolate was referred to GBRU for confirmation in November 2017. It was grown on selective media, MacConkey and chromogenic agar, to rule out contamination from other Enterobacteriaceae. A single colony was selected for inoculation into broth for WGS DNA extraction and grown in Mueller-Hinton agar for antimicrobial susceptibility testing. Ethical approval for the detection of gastrointestinal bacterial pathogens from faecal specimens, or the identification, characterization and typing of cultures of gastrointestinal pathogens, submitted to GBRU is not required as covered by PHE’s surveillance mandate.

### Antimicrobial susceptibility testing

Minimal inhibitory concentration (MICs) of the isolate were determined by agar dilution using Mueller–Hinton agar for the standard panel of antibiotics recommend by EUCAST. EUCAST breakpoints and screening concentration criteria were used for interpretation [21]. Confirmation of azithromycin MIC was performed by Etest^VR^ (bioMerieux, France). Temocillin and cefoxitin were included in the panel to aid detection of OXA-48-like carbapenemases and AmpC production, respectively. ESBL detection was confirmed using aztreonam, cefotaxime/cefotaxime + clavulanic acid (4ug/mL, ceftazidime/ceftazidime + clavulanic acid (4ug/mL), cefepime/cefepime+clavulanic acid (4ug/mL).

### Whole genome sequencing and analysis

DNA extraction of the *Salmonella* isolate was carried out using a modified protocol of the Qiasymphony DSP DNA Midi Kit (Qiagen) as described in Nair *et al*. 2016 [22]. In brief, 0.7 mL of overnight *Salmonella* broth culture was harvested. Bacterial cells were pre-lysed in 220 μL of ATL buffer (Qiagen) and 20 μL of Proteinase K (Qiagen), and incubated with shaking for 30 min at 56°C. Four microlitres of RNase at 100 mg/mL (Qiagen) was added to the lysed cells, which were then re-incubated for a further 15 min at 37°C. DNA from the treated cells was then extracted on the Qiasymphony SP platform (Qiagen) and eluted in 100 μL of water. Extracted DNA was fragmented and tagged for multiplexing with NEXTERA XT DNA Sample Preparation Kits, followed by paired-end sequencing on an Illumina HiSeq platform to produce 101 bp paired-end reads (Illumina, Cambridge, UK).

Resistance genes were determined using Genefinder, a customised algorithm that uses Bowtie 2 to map reads to a set of reference sequences and Samtools to generate an mpileup file [23], as previously described Day *et al*. 2018 [3]. Briefly, the data are parsed based on read coverage of the query sequence (100%), consensus base-call on variation (>85%) and the nucleotide identity (>90%) to determine the presence of the reference sequence or nucleotide variation within that sequence. β-Lactamase variants were determined with 100% identity using the reference sequences downloaded from the Lahey (www.lahey.org) or NCBI (https://www.ncbi.nlm.nih.gov/pathogens/beta-lactamase-data-resources) β-lactamase data resources. Known acquired resistance genes and resistance-conferring mutations relevant to β-lactams, fluroquinolones, aminoglycosides, chloramphenicol, macrolides, sulphanomides, tetracyclines, trimethoprim, rifamycins and Fosfomycin were included in the analysis [24][25].

Sequence type (ST), eBurst Group (eBG) and serovar were determined from the genome data using MOST v1.0 as previously described [26][27].

PlasmidFinder v2.1 (http://cge.cbs.dtu.dk/services/PlasmidFinder/) was used to detect the presence of known replicon types of plasmids in the isolates studied [28].

### Location and characterization of region encoding ß-lactam resistance

*De novo* assembly graphs (in FASTG format) produced by Spades v3.7.0 were visualized using Bandage v0.8.1 (http://github.com/rrwick/Bandage) [29]. BLAST analysis (blast.ncbi.nlm.nih.gov/Blast.cgi) was conducted to detect the antimicrobial resistance genes (*bla*_BLACTX-M-15_ and the ISEcp9 mobile insertion sequence) and their location in the assembly graph. Comparisons with previously described IncI1 plasmids associated with ß-lactam resistance from *S*. Typhi [pPRJEB21992](EMBL-EBI BioProject PRJEB21992) and *S*. Enteritidis [pSE115] (GenBank accession number KT868530) [30][31] were also undertaken with BLAST. Prokka v1.12 was used to annotate genome sequences (http://www.ncbi.nlm.nih.gov/pubmed/24642063) [32] and Artemis v18.00 (www.sanger.ac.uk/resources/software/artemis) used to visualize the resistance region. Default settings were used for all the bioinformatics tools used in this study.

#### Pairwise BLAST comparison of IncI1 plasmids harbouring ß-lactam resistance

Plasmids from *S*. Typhi (BioProject PRJEB21992) [30], S. Enteritidis (KT868530) [31] and *S*. Enteritidis (accession NC_018659) were selected for comparison using the following two criteria: (i) the presence of the same mobile element and resistance gene (ISEcp9 and (*bla*_BLACTX-M-15_) and (ii) the same IncI1 incompatibility group as the plasmid from isolate 440915 being investigated. FASTA files from all plasmids were compared and visualised using BRIG v0.95 [33]. The plasmid fasta was extracted for pPRJEB21992 from a plasmid SPAdes assembly performed on the genome sequence data from accession PRJEB21992 [34] (version 3.11.1, ‘-careful’). The orientation and position of specific genes was drawn using Easyfig v 2.1 [35].

#### Nucleotide sequence accession number

Short-read FASTQ sequence for the *S*. Paratyphi A 440915 plasmid described in this study has been deposited in the NCBI Sequence Read Archive under GenBank accession number MK238490 and BioProject PRJNA505238.

#### Phylogenetic analysis

To place isolate 440915 in context of the *S*. Paratyphi A population, SNP analysis was performed on the 439 isolates of *S*. Paratyphi A referred to PHE from 1st April 2014 to December 2017 (S1 Table). The PHE isolates were supplemented with 20 genome sequences from *Zhou et al*. 2018 [36] with representatives covering the 7 described lineages (A-G). Illumina reads were quality trimmed [37] with bases removed from the forward and trailing end with a PHRED score of less than 30. Reads were mapped to the *S*. Paratyphi A reference genome ATCC 9150 (Genbank accession CP000026.1) using BWA-MEM v.0.7.12 [38]. SNPs were identified using GATK v.2.6.5 [39] in unified genotyper mode. Core genome positions that had a highquality SNP (>90% consensus, minimum depth 10x, GQ> = 30) in at least one isolate were extracted using SnapperDB v0.2.5 [40] and processed through Gubbins v2.0.0 [41] to account for recombination events. RaxML v.8.1.17 [42] was used to derive the maximum likelihood phylogeny of the isolates using the GTRCAT substitution model with the automatic bootstrapping criteria ‘autoMRE’. FASTQ reads from all sequences in this study can be found at the PHE Pathogens BioProject at the National Centre for Biotechnology Information (Accession PRJNA248792).

### WGS process

The whole WGS process, from growing bacterial cells to interpreting sequence data for identification, AMR characterisation and high throughput single nucleotide polymorphism typing for surveillance requires approximately 5 days. A rapid turnaround time considering the amount of data obtained.

## Results

A 44 year old male patient who had returned to England from a 6 week trip to Bangladesh in September 2017 was diagnosed with infective colitis. From a stool specimen, an enteric PCR was positive for *Salmonella* spp; the stool cultured a presumptive *S*. Paratyphi A that was resistant to quinolones, and sensitive to azithromycin. The isolate was sent to GBRU and confirmed as *S*. Paratyphi A (isolate 440915) in November 2017 by WGS. Further case details are given in Supplementary Data (S1 Data).

Our WGS analysis revealed *S*. Paratyphi A 440915 to be an ESBL-producing strain encoding both *bla*_BLACTX-M-15_ and a *bla*_TEM-191_. A point mutation conferring resistance to quinolones was detected in *gyrA* [83:S-F].ESBL production was phenotypically confirmed with cefotaxime clavulanic acid synergy. The ciprofloxacin MIC was 0.75 mg/L and the isolate was sensitive to azithromycin (8 mg/L).

**Fig 1:**
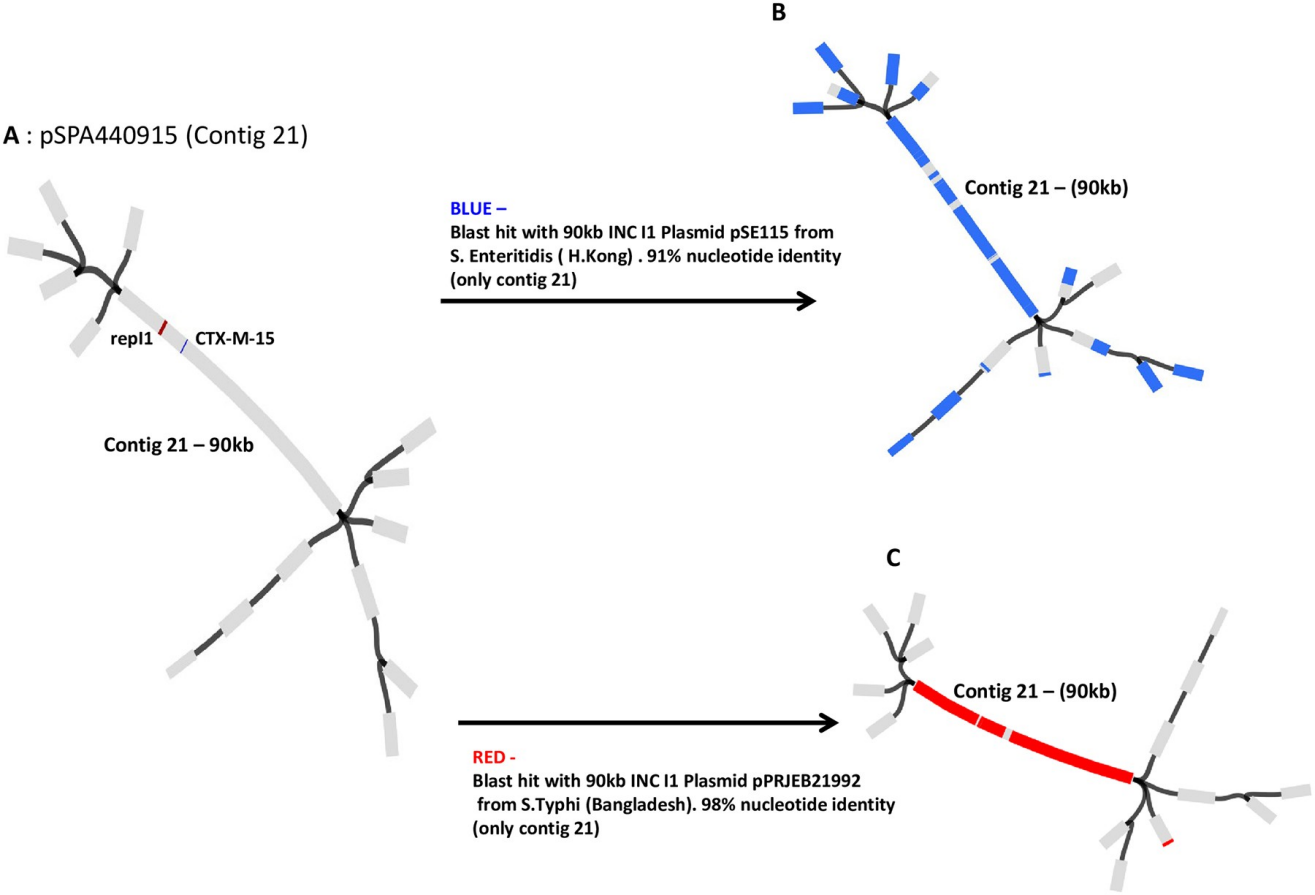
Bandage analysis of the resistant *S*. Paratyphi A 440915 isolate. A SPAdes assembly of the sequence regions (contigs) associated with drug resistance from isolate 440915. Bandage allows visualization of how contigs (in gray) are possibly connected (in black) to each other. (A) ~90kb plasmid pSPA440915 (contig 21) was assembled from the complete genome sequence of *S*. Paratyphi A 440915.ESBL resistant *bla*_CTX-M-15_ gene (blue) and repI1 indicating IncI1 plasmid replicon were blasted against the assembled pSPA440915, and their location determined (on contig 21). (B) Plasmid pSE115 from *S*. Enteritidis (Gene Bank accession number: KT868530) was blasted against pSPA440915. Blast hits in blue (only in contig 21) indicates a 91% sequence similarity between both plasmids. repI1 present. *bla*_CTX-M-14_ present instead of a *bla*_CTX-M-15_ gene. Blast Hits (blue) outside of contig 21 –mainly short repeat sequences (insertion elements). (C) Plasmid pPRJEB21992 from *S*. Typhi (EMBL-EBI BioProject PRJEB21992) was blasted against pSPA440915. Blast hits in red (only in contig 21) indicates a 98% sequence similarity between both plasmids, including the *repI1*, *bla*_CTX-M-15_ and *bla*_TEM-191_.

Both Bandage and BLAST analysis confirmed the *bla*_CTX-M-15_, *bla*_TEM -191_ and repI1 (IncI1 plasmid replicon) genes to be present on a 90 kb contig, subsequently confirmed using PlasmidFinder to be an IncI1 plasmid (Fig 1A). Plasmid pSPA440915 was highly similar to two previously reported ~90kb IncI1 plasmids: *S*. Enteritidis plasmid pSE115 (91% nucleotide identity) isolated in Hong Kong [31] (Fig 1B) and *S*. Typhi plasmid pPRJEB21992 (98% identity) from Bangladesh [30] (Fig1C).

**Fig 2:**
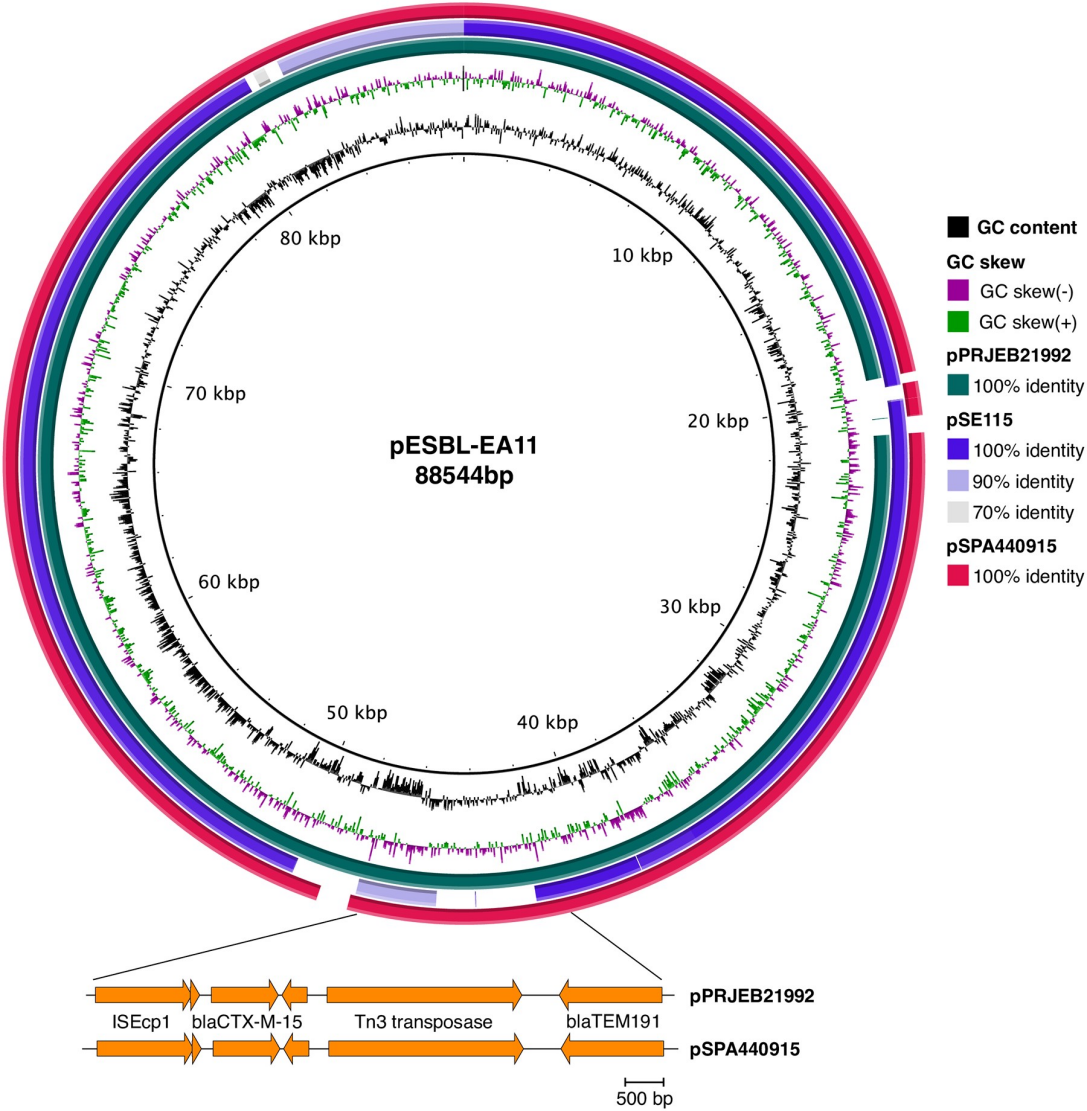
Pairwise BLAST comparisons of IncI1 plasmids against *E*. *coli* plasmid pESBL_EA11 (inner ring, black line) generated using BRIG [33]. Ring 2 and 3 represent GC content (black) and GC skew (purple/green). Ring 3 (teal): *S*. Typhi plasmid pPRJEB21992; ring 4 (purple): *S*. Enteritidis plasmid pSE115; ring 5 (red): *S*. Paratyphi A plasmid pSPA440915. Expanded region (genes in orange) indicating presence of beta lactamases, drawn using Easyfig [35]. Presence of ESBL mobile drug cassette ISEcp1, *bla*_CTX-M-15_ and *bla*_191_ in both the Bangladesh *S*. Typhi and *S*. Paratyphi A 440915 isolate.

The ESBL mobile drug cassette ISEcp9-_*bla*CTX-M-15_-hp-tnpA with an additional *bla*_TEM -191_ was identified in the plasmids belonging to *S*. Typhi PRJEB21992 and *S*. Paratyphi A 440915, both from Bangladesh (Figs 1A, 1C and 2). A different ESBL mobile element, ISEcp9-*bla*_CTX-M-14_-tnpA was mapped to the *S*. Enteritidis plasmid pSE115 (Fig 2).

**Fig 3:**
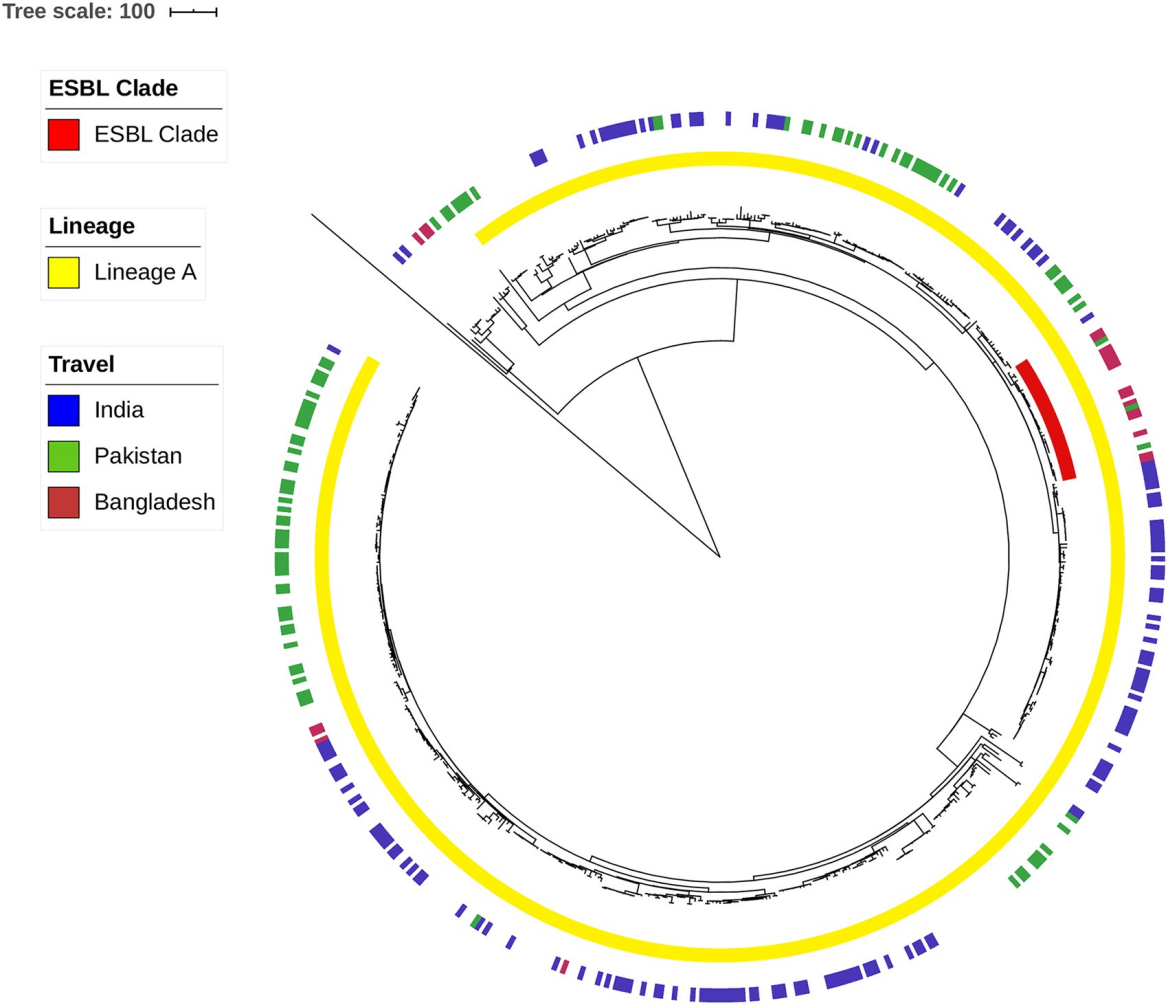
Recombination free maximum likelihood phylogeny of 459 isolates including 20 reference isolates from Zhou *et al*. 2014 [36]. Middle ring (yellow) represents isolates that cluster into lineage A. Inner ring (red) represents the clade isolate 440915 clusters in. Outer ring represents country of travel for UK cases (Burgundy–Bangladesh, Blue–India, Green–Pakistan).

Isolate 440915 was confirmed as *S*. Paratyphi A multi locus sequence type (ST)129, a member of serovar Paratyphi A eBURST group 11. *S*. Paratyphi A 440915 clusters in lineage A of the *S*. Paratyphi A population as defined by Zhou *et al*. 2014 [36]. Within lineage A, isolate 440915 belongs to a monophyletic clade with 29 other isolates from cases reported through GBRUs routine surveillance. Of these cases 16/29 (55%) reported recent travel to Bangladesh, 2/29 reported travel to Pakistan, 1/29 reported no travel and the remaining 10 cases had no travel information available. Within this clade all isolates harboured a single mutation in *gyrA* [83:S-F] conferring resistance to ciprofloxacin; no other resistant determinants were detected (Fig 3).

## Discussion

This study reports the first ESBL-producing *S*. Paratyphi A isolated in the UK using PHE’s WGS-based surveillance for AMR determinants [3][19]

CTX-M enzymes have emerged and disseminated worldwide since the early 2000s and have become the dominant ESBLs in Enterobacteriaceae in both hospitals and community settings [43][44]. It is unsurprising that isolate 440915 harboured *bla*_CTX-M-15_ on an IncI1 plasmid; these plasmids have been most frequently associated with *bla*_CTX-M_ type ESBL carriage [45], are transmissible between enteric pathogens [46] and commonly found in the Enterobacteriaceae. IncI1 plasmids have also been shown to have no biological cost on the fitness of *E*. *coli* isolates harbouring them [47] which means they may be maintained even in the absence of selective antibiotic pressure. The high nucleotide identity between ISEcp9-*bla*_CTX-M-15_-hp-tnpA in *S*. Typhi PRJEB21992 [30] and the current *S*. Paratyphi A 440915 isolate in this study makes it plausible to speculate that one transmitted to the other and hence supports the transmissible nature of ISEcp9 linked to ß-lactam resistance [48].

ESBL-carrying *S*. Paratyphi A and *S*. Typhi isolates were not seen globally or particularly in Europe until recently [10][13][49][50]. The ESBL *S*. Typhi isolate recently reported from Bangladesh was the second isolate since 2000 [30]. A global population structure study by Zhou *et al*. 2018 [36] classified *S*. Paratyphi A into seven lineages with lineages A and C being the most dominant. The phylogenetic analysis described here indicates that *S*. Paratyphi A 440915 is closely related to *S*. Paratyphi A belonging to lineage A, circulating in Bangladesh and the Indian subcontinent (Fig 3). We therefore postulate that the dissemination of ESBL resistance can be sustained by different mechanisms including the horizontal dissemination of this transferable IncI1 plasmid or transposition of the mobile element (ISEcp9- *bla*_CTX-M-15_-hp-tnpA) within this closely related lineage. We believe it is only a matter of time until the ISEcp9 *bla*_CTX-M-15_-hp-tnpA mobile element (or a variation) inserts into a successful chromosomal background, as seen with the recent outbreak in Pakistan caused by *S*. Typhi H58 and an IncY plasmid [13].

This ESBL *S*. Paratyphi A 440915 isolate and recent sporadic and outbreak ESBL enteric fever cases reported in the UK, Germany and Pakistan [13][49][50] indicate the need for active surveillance in the UK for cases returning from the Indian subcontinent. In the UK, the standard empirical treatment for complicated enteric fever from South Asia has continued to be third-generation cephalosporins. Diagnostic laboratories in England usually perform AST on faecal isolates of typhoidal salmonellae with antibiotics such as azithromycin and ciprofloxacin, using gradient assays like E-tests (bioMerieux, France). However, in order to avoid treatment failures, AST should be routinely performed accordingly to EUCAST guidelines on all presumptive isolates (faecal and invasive) of *S*. Paratyphi and *S*. Typhi specifically looking for ceftriaxone resistance. Even though WGS predicts AMR determinants it is not used for clinical management and hence ceftriaxone resistant strains are phenotypically tested to assess ESBL production [51] to tailor clinical treatment. Treatment should be tailored depending on the AST. The patient in this case fully recovered from the illness and had negative clearance specimens one year later despite not being treated with the appropriate antibiotics (refer to S1 Data). Although paratyphoid is a milder illness than typhoid fever, further research is required to monitor outcomes of ESBL producing strains including enhanced clinical surveillance to assess whether the clinical outcome includes relapses.

## Conclusion

Extended spectrum ß-lactamase producing *S*. Paratyphi A has been identified in the UK from the Indian subcontinent through PHE’s use of routine WGS. Routine WGS provides a rapid and accurate method for surveillance of drug resistance genes and can inform the national treatment guidelines for management of enteric fever. WGS data as obtained by GBRU also allows passive surveillance to monitor the spread of drug resistant *S*. Typhi and *S*. Paratyphi and to detect outbreaks, as well as to serve as a sentinel surveillance for drug resistant enteric fever agents circulating in different regions of the world where the disease is endemic.

## Supporting information

S1 Table Isolates that were SNP analysed. (XLSX)

S1 Data Case history of patient. (DOCX)

## References

[1] Crump JA, Mintz ED. Global trends in typhoid and paratyphoid fever. *CID* 2010; 50:241–245. 10.1086/649541 PMC2798017 20014951

[2] Threlfall EJ, Fisher IS, Berghold C, Gerner-Smidt P, Tschape H, Cormican M, et al Trends in antimicrobial drug resistance in Europe, 1999–2001. *Int J Antimicrobial Agents* 2003; 22:487–91. 10.1016/s0924-8579(03)00262-0 14602366

[3] Day RM, Doumith M, Do Nascimento V, Nair S, Ashton PM, Jenkins C, et al Comparison of phenotypic and WGS-derived antimicrobial resistance. profiles of *Salmonella enterica* serovars Typhi and Paratyphi. *J Antimicrob Chemother* 2018; 73: 365–372. 10.1093/jac/dkx379 29216342

[4] European Centre for Disease Prevention and Control. Typhoid and paratyphoid fever. In:ECDC. Annual Epidemiological report for 2015. Stockholm: ECDC;2018. https://ecdc.europa.eu/en/publications-data/typhoid-and-paratyphoid-fever-annual-epidemiological-report-2015#no-link

[5] Mohanty S, Renuka K, Sood S, Das BK, Kapil A. Antibiogram pattern and seasonality of *Salmonella* serotypes in a North Indian tertiary care hospital. *Epidemiol Infect* 2006 134:961–966. 10.1017/S0950268805005844 16476168 PMC2870474

[6] Woods CW, Murdoch DR, Zimmerman MD, Glover WA, Basnyat B, Wolf L, et al Emergence of *Salmonella enterica* serotype Paratyphi A as a major cause of enteric fever in Kathmandu, Nepal. *Trans R Soc Trop Med Hyg* 2006; 100:1063–1067. 10.1016/j.trstmh.2005.12.011 16714040

[7] Maskey AP, Basnyat B, Thwaites GE, Campbell JI, Farrar JJ, Zimmerman MD. Emerging trends in enteric fever in Nepal: 9124 cases confirmed by blood culture 1993–2003. *Trans R Soc Trop Med Hyg* 2008; 102(1):91–5. 10.1016/j.trstmh.2007.10.003 18023462

[8] Adachi T, Sagara H, Hirose K, Watanabe H. Fluoroquinolone-resistant *Salmonella* Paratyphi A. *Emerg Infect Dis* 2005; 11:172–174. 10.3201/eid1101.040145 15714664 PMC3294367

[9] Wain J, Hendriksen RS, Mikoleit ML, Keddy K, Ochiai RL. Typhoid fever. *Lancet* 2015; 385:1136–45. 10.1016/S0140-6736(13)62708-7 25458731 PMC11567078

[10] Mawatari M, Kato Y, Hayakawa K, Morita M, Yamada K, Mezaki K, et al *Salmonella enterica* serotype Paratyphi A carrying *bla*_CTX-M-15_ type extended-spectrum beta-lactamase isolated from a Japanese traveller returning from India to Japan, *Euro Surveill* 2013;18(46). Available online: http://www.eurosurveillance.org/ViewArticle.aspx?ArticleId=20632 10.2807/1560-7917.es2013.18.46.20632 24256887

[11] Molloy A, Nair S, Cooke FJ, Wain J, Farrington M, Lehner PJ, et al First report of *Salmonella enterica* serotype Paratyphi A azithromycin resistance leading to treatment failure. *J Clin Microbiol* 2010; 48(12):4655–7. 10.1128/JCM.00648-10 20943875 PMC3008474

[12] Ahsan S, Rahman S. Azithromycin Resistance in Clinical Isolates of *Salmonella enterica* Serovars Typhi and Paratyphi in Bangladesh. *Microbial Drug Resis* 2019; 25:8–13. 10.1089/mdr.2018.0109 30016183

[13] Klemm EJ, Shakoor S, Page AJ, Qamar FN, Judge K, Saeed DK, et al Emergence of an Extensively Drug-Resistant *Salmonella enterica* Serovar Typhi Clone Harboring a Promiscuous Plasmid Encoding Resistance to Fluoroquinolones and Third-Generation Cephalosporins. *mBIO* 2018; 9:e00105–18. 10.1128/mBio.00105-18 29463654 PMC5821095

[14] Su LH, Chiu CH, Chu C, Ou JT. Antimicrobial resistance in nontyphoid *Salmonella* serotypes: a global challenge. *Clin Infect Dis* 2004; 39: 546–551. 10.1086/422726 15356819

[15] Hopkins KL, Liebana E, Villa L, Batchelor M, Threlfall EJ, Carattoli A. Replicon typing of plasmids carrying CTX-M or CMY beta lactamases circulating among *Salmonella* and *Escherichia coli* isolates. *Antimicrob Agents Chemother* 2006; 50:3203–3206. 10.1128/AAC.00149-06 16940132 PMC1563510

[16] Jean SS, Hsueh PR. High burden of antimicrobial resistance in Asia. *Int J Antimicrob Agents* 2011; 37:291–295. 10.1016/j.ijantimicag.2011.01.009, 21382699

[17] Xia S, Hendriksen RS, Xie Z, Huang L, Zhang J, Guo W, et al Molecular characterization and antimicrobial susceptibility of *Salmonella* isolates from infections in humans in Henan Province, China. *J Clin Microbiol* 2009; 47:401–409. 10.1128/JCM.01099-08 19073874 PMC2643658

[18] Carattoli A. Resistance plasmid families in Enterobacteriaceae. *Antimicrob Agents Chemother* 2009; 53:2227–2238. 10.1128/AAC.01707-08 19307361 PMC2687249

[19] Ashton PM, Nair S, Peters T, Bale J, Powell D, Painset A, et al Identification of *Salmonella* for public health surveillance using whole genome sequencing. *PeerJ* 2016; 4:e1752 10.7717/peerj.1752 27069781 PMC4824889

[20] Neuert S, Nair S, Day MR, Doumith M, Ashton PM, Mellor KC, et al Prediction of phenotypic antimicrobial resistance profiles from whole genome sequences of non-typhoidal *Salmonella enterica*. *Front Microbiol* 2018 27; 9:592 10.3389/fmicb.2018.00592 eCollection 2018. 29636749 PMC5880904

[21] The European Committee on Antimicrobial Susceptibility Testing. Routine and extended internal quality control for MIC determination and disk diffusion as recommended by EUCAST. Version 9.0. 2019; http://www.eucast.org

[22] Nair S, Ashton P, Doumith M, Connell S, Painset A, Mwaigwisya S, et al WGS for surveillance of antimicrobial resistance: a pilot study to detect the prevalence and mechanism of resistance to azithromycin in a UK population of non-typhoidal *Salmonella*. *J Antimicrobial Chemother* 2016; 71(12):3400–3408. 10.1093/jac/dkw318 27585964

[23] Langmead B, Salzberg SL. Fast gapped-read alignment with Bowtie 2. *Nat Methods* 2012; 9:357–9. 10.1038/nmeth.1923 22388286 PMC3322381

[24] Day M, Doumith M, Jenkins CD, Dallman TJ, Hopkins KL, Elson R, et al Antimicrobial resistance in Shiga toxin-producing *Escherichia coli* serogroups O157 and O26 isolated from human cases of diarrhoeal disease in England, 2015. *J Antimicrob Chemother* 2017; 72:145–52. 10.1093/jac/dkw371 27678285

[25] Sadouki Z, Day MR, Doumith M, Chattaway MA, Dallman TJ, Hopkins KL, et al Comparison of phenotypic and WGS derived antimicrobial resistance profiles of *Shigella sonnei* isolated from cases of diarrhoeal disease in England and Wales 2015. *J Antimicrob Chemother* 2017; 72:2496–502. 10.1093/jac/dkx170 28591819

[26] Tewolde R, Dallman T, Schaefer U, Sheppard CL, Ashton P, Pichon B, et al MOST: a modified MLST typing tool based on short read sequencing. *Peer J* 2016; 4:e2308 10.7717/peerj.2308 27602279 PMC4991843

[27] Achtman M, Wain J, Weill FX, Nair S, Zhou Z, Sangal V, et al Multilocus sequence typing as a replacement for serotyping in *Salmonella* enterica. *PLoS Pathog* 2012; 8: e1002776 10.1371/journal.ppat.1002776 22737074 PMC3380943

[28] Carattoli A, Zankari E, Garcıa-Fernandez A, Voldby Larsen M, Lund O, Villa L, et al In silico detection and typing of plasmids using PlasmidFinder and plasmid multilocus sequence typing. *Antimicrob Agents Chemother* 2014; 58: 3895–903. 10.1128/AAC.02412-14 24777092 PMC4068535

[29] Wicks RR, Schultz MB, Zobel J, Holt KE. Bandage: interactive visualisation of de novo genome assemblies. *Bioinformatics* 2015; 31: 3350–2. 10.1093/bioinformatics/btv383 26099265 PMC4595904

[30] Djeghout B, Saha S, Sajib MSI, Tanmoy AM, Islam M, Kay GL, et al Ceftriaxone-resistant *Salmonella* Typhi carries an IncI1-ST31 plasmid encoding BLACTX-M-15. *J Med Microbiol* 2018; 10.1099/jmm.0.000727 29616895

[31] Wong MH, Biao K, Cjan EW, Yan M, Chen S. IncI1 plasmids carrying various *bla*_CTX-M_ genes contribute to ceftriaxone resistance in *Salmonella enterica* Serovar Enteritidis in China. *Antimicrobial Agents Chemother* 2016; 60:982–989. 10.1128/AAC.02746-15 26643327 PMC4750657

[32] Seeman T. Prokka:rapid prokaryotic genome annotation. *Bioinformatics* 2014; 30: 2068–9. 10.1093/bioinformatics/btu153 24642063

[33] Alikhan NF, Petty NK, Ben Zakour NL, Beatson SA. BLAST Ring Image Generator (BRIG): simple prokaryote genome comparisons. *BMC Genomics* 2011; 12:402 10.1186/1471-2164-12-402 21824423 PMC3163573

[34] Nurk S, Bankevich A, Antipov D, Gurevich AA, Korobeynikov A, Lapidus A, et al Assembling single-cell genomes and mini-metagenomes from chimeric MDA products. *J Computational Biol* 2013: 20(10):714–737. 10.1089/cmb.2013.0084 PMC3791033 24093227

[35] Sullivan MJ, Petty NK, Beatson SA. Easyfig: a genome comparison visualiser. *Bioinformatics* 2011; 27: 1009–10. 10.1093/bioinformatics/btr039 21278367 PMC3065679

[36] Zhou Z, McCann A, Weill FX, Blin C, Nair S, Wain J, et al Transient Darwinian selection in *Salmonella enterica* serovar Paratyphi A during 450 years of global spread of enteric fever. *PNAS* 2014; 11:12199–12204. 10.1073/pnas.1411012111 PMC4143038 25092320

[37] Bolger AM, Lohse M, Usadel B. Trimmomatic: A flexible trimmer for Illumina Sequence Data. *Bioinformatics* 2014; 30(15):2114–20. 10.1093/bioinformatics/btu170 24695404 PMC4103590

[38] Li H, Durbin R. Fast and accurate long-read alignment with Burrows-Wheeler transform. *Bioinformatics* 2010; 26(5):589–95. 10.1093/bioinformatics/btp698 Epub 2010 Jan 15 20080505 PMC2828108

[39] McKenna A, Hanna M, Banks E, Sivachenko A, Cibulskis K, Kernytsky A, et al The Genome Analysis Toolkit: a MapReduce frame- work for analyzing next-generation DNA sequencing data. *Genome Res* 2010; 20(9):1297–303. 10.1101/gr.107524.110 20644199 PMC2928508

[40] Dallman T, Ashton P, Schafer U, Jironkin A, Painset A, Shaaban S, et al SnapperDB: A database solution for routine sequencing analysis of bacterial isolates. *Bioinformatics* 2018; 10.1093/bioinformatics/bty212 29659710

[41] Croucher NJ, Page AJ, Connor TR, Delaney AJ, Keane JA, Bentley SD, et al Rapid phylogenetic analysis of large samples of recombinant bacterial whole genome sequences using Gubbins. *Nucleic Acids Res* 2014; 43(3):e15 10.1093/nar/gku1196 25414349 PMC4330336

[42] Stamatakis A. RAxML Version 8: A tool for Phylogenetic Analysis and Post-Analysis of Large Phylogenies. *Bioinformatics* 2014; 30(9):1312–13. 10.1093/bioinformatics/btu033 24451623 PMC3998144

[43] D’Andrea MM, Arena F, Pallecchi L, Rossolini GM. CTX-M-type -lactamases: a successful story of antibiotic resistance. *Int J Med Microbiol* 2013; 303:305–317. 10.1016/j.ijmm.2013.02.008 23490927

[44] Cantón R, González-Alba JM, Galán JC. CTX-M enzymes: origin and diffusion. *Front Microbiol* 2012; 3:110 10.3389/fmicb.00110 22485109 PMC3316993

[45] Carattoli A. Plasmids and the spread of resistance. *Int J Med Microbiol* 2013; 303:298–304. 10.1016/j.ijmm.2013.02.001 23499304

[46] Rozwandowicz M, Brouwer MSM, Fischer J, Wagenaar JA, Gonzalez-Zorn B, Guerra B, et al Plasmids carrying antimicrobial resistance genes in Enterobacteriaceae. *J Antimicrob Chemother* 2018; 73(5):1121–1137. 10.1093/jac/dkx488 29370371

[47] Riccobono E, Di Pilato V, Di Maggio T, Revollo C, Bartoloni A, Pallecchi L, et al Characterization of IncI1 sequence type 71 epidemic plasmid lineage responsible for the recent dissemination of CTX-M-65 extended-spectrum -lactamase in the Bolivian Chaco region. *Antimicrob Agents Chemother* 2015; 59:5340–5347. 10.1128/AAC.00589-15 26100713 PMC4538553

[48] Poirel L, Decousser JW, Nordmann P. Insertion sequence ISEcp1B is involved in expression and mobilization of a *bla*_CTX-M_ b-lactamase gene. *Antimicrob Agents Chemother* 2003; 47:2938–2945. 10.1128/AAC.47.9.2938-2945.2003 12936998 PMC182628

[49] Kleine CE, Schlabe S, Hischebeth GTR, Molitor E, Pfeifer Y, Wasmuth JC, et al Successful therapy of a multidrug-resistant extended-spectrum β-lactamase–producing and fluoroquinolone-resistant *Salmonella enterica* Subspecies enterica serovar Typhi infection using combination therapy of meropenem and fosfomycin. *Clin Infect Dis* 2017; 65:1754–6. 10.1093/cid/cix652 29020162

[50] Godbole GS, Day MR, Murthy S, Chattaway MA, Nair S. First report of BLACTX-M-15 *Salmonella Typhi* from England. *Clin Infect Dis* 2018; 66(12):1976–1977. 10.1093/cid/ciy032 29471386

[51] Ellington MJ, Ekelund O, Aarestrup FM, Canton R, Doumith M, Giske C, et al The role of whole genome sequencing in antimicrobial susceptibility testing of bacteria: report from the EUCAST Subcommittee. *Clin Microbiol Infect* 2017; 23(1):2–22. 10.1016/j.cmi.2016.11.012 Epub 2016 Nov 23. 27890457

