# Supplementary material for: Genomic surveillance detects Salmonella enterica serovar Paratyphi A harbouring blaCTX-M-15 from a traveller returning from Bangladesh
Source: PLoS One. 2020 Jan 30;15(1):e0228250. doi: 10.1371/journal.pone.0228250 (PMC6992414; doi:10.1371/journal.pone.0228250)
Supplement: S1 Data — (DOCX) [file pone.0228250.s002.docx]

Supplementary Data 1

A review of the case history confirmed the patient was a 44 year old adult male, who had returned to England 2 weeks prior, from a 6 week trip to Bangladesh in September 2017. He presented to his local hospital in September 2017 two weeks after his return, with a history of 2 days of fever and diarrhoea. He was born in Bangladesh and had lived there for first 20 years of his life before moving to England. He was diagnosed with infective colitis and empirically commenced with IV ceftriaxone and oral metronidazole. He was discharged home after 48 hours with a course of oral ciprofloxacin. From a stool specimen, an enteric PCR was positive for *Salmonella* spp; the stool cultured a presumptive *S*. Paratyphi A that was resistant to quinolones, and sensitive to azithromycin. Ceftriaxone susceptibility were not performed at the local hospital. The isolate (440915) was later sent to GBRU and confirmed as *S*. Paratyphi A in November 2017 by WGS.

The patient returned to hospital 4 weeks later with a history of intermittent fever and vomiting; he had no further diarrhoea. His full blood count was normal but he had transaminitis with ALT raised at 327 IU/L (normal 0-40), alkaline phosphatase 183 IU/L (normal 20-130 IU/L), bilirubin was 15 umol/L (normal <19 umol/L). The malaria, dengue and hepatitis A, B, C screen was negative, though there was evidence of previous hepatitis E (IgG positive). An ultrasound of the liver including biliary tract and gall bladder was normal; no gall stones were seen. The stool was PCR and culture negative for *Salmonella* spp. The patient was recommenced on ceftriaxone as partially treated enteric fever and 14 days of treatment were completed. Blood cultures remained negative, and both malaria and HIV screens were negative.

At the one year follow up, the patient was well and had fully recovered with mildly elevated liver functions (ALT 53 IU/L) and normal full blood count. Both the stool PCR and culture were negative for *Salmonella* spp.
